# Lack of Spleen Signal on Diffusion Weighted MRI is associated with High Tumor Burden and Poor Prognosis in Multiple Myeloma: A Link to Extramedullary Hematopoiesis?

**DOI:** 10.7150/thno.33289

**Published:** 2019-07-09

**Authors:** Leo Rasche, Manoj Kumar, Grant Gershner, Rohan Samant, Rudy Van Hemert, Anke Heidemeier, Constantin Lapa, Thorsten Bley, Andreas Buck, James McDonald, Jens Hillengass, Joshua Epstein, Sharmilan Thanendrarajan, Carolina Schinke, Frits van Rhee, Maurizio Zangari, Bart Barlogie, Faith E Davies, Gareth J Morgan, Niels Weinhold

**Affiliations:** 1 Myeloma Center, University of Arkansas for Medical Sciences, Little Rock, AR, USA; 2 Department of Internal Medicine II, University Hospital of Würzburg, Würzburg, Germany; 3 Radiology Department, University of Arkansas for Medical Sciences, Little Rock, AR, USA; 4 Radiology Department, University Hospital of Würzburg, Würzburg, Germany; 5 Department of Nuclear Medicine, University Hospital of Würzburg, Würzburg, Germany; 6 Roswell Park Comprehensive Cancer Center, Buffalo, NY, USA; 7 Department of Internal Medicine V, University Hospital of Heidelberg, Heidelberg, Germany

**Keywords:** multiple myeloma, diffusion weighted mri, spleen, tumor burden, high risk, extramedullary hematopoiesis

## Abstract

Due to the low frequency of abnormalities affecting the spleen, this organ is often overlooked during radiological examinations. Here, we report on the unexpected finding, that the spleen signal on diffusion-weighted MRI (DW-MRI) is associated with clinical parameters in patients with plasma cell dyscrasias.

**Methods**: We investigated the spleen signal on DW-MRI together with clinical and molecular parameters in 295 transplant-eligible newly diagnosed Multiple Myeloma (NDMM) patients and in 72 cases with monoclonal gammopathy of undetermined significance (MGUS).

**Results**: Usually, the spleen is the abdominal organ with the highest intensities on DW-MRI. Yet, significant signal loss on DW-MRI images was seen in 71 of 295 (24%) NDMM patients. This phenomenon was associated with the level of bone marrow plasmacytosis (*P*=1x10^-10^) and International Staging System 3 (*P*=0.0001) but not with gain(1q), and del(17p) or plasma cell gene signatures. The signal was preserved in 72 individuals with monoclonal gammopathy of undetermined significance and generally re-appeared in MM patients responding to treatment, suggesting that lack of signal reflects increased tumor burden. While absence of spleen signal in MM patients with high risk disease defined a subgroup with very poor outcome, re-appearance of the spleen signal after autologous stem cell transplantation was seen in patients with improved outcome. Our preliminary observation suggests that extramedullary hematopoiesis in the spleen is a factor that modifies the DW-MRI signal of this organ.

**Conclusions**: The DW-MRI spleen signal is a promising marker for tumor load and provides prognostic information in MM.

## Introduction

Whole body imaging is an integral component of Multiple Myeloma (MM) evaluation as it reveals bone disease and tumor infiltration 1. While established techniques, such as ^18^F-Fluorodeoxyglucose (FDG)- positron emission tomography computer tomography (PET-CT), are powerful in delineating focal lesions (FLs) 2-5, they suffer from low specificity when it comes to assessment of diffuse bone marrow (BM) signals, which originate from interstitial MM growth. Diffusion-weighted MRI with background suppression (DW-MRI) is a functional technique, which measures water diffusion *in vivo* 6. As diffusion of water is more restricted in tissues with high cellularity, DW-MRI is a promising technique for investigating diffuse BM signals. To classify the intensity of the BM signal in MM patients using DW-MRI, we selected the spleen as internal reference, since it is the abdominal organ with the highest restriction 7. Unexpectedly, we observed significant heterogeneity in the spleen signal itself including a subgroup of MM patients with total lack of spleen visualization. Lack of signal was primarily seen in patients with hyperintense diffuse BM signals, suggesting that the spleen intensity could be a candidate surrogate marker for tumor load in MM. Here, we report on our attempt to elucidate this phenomenon and to evaluate its clinical value.

## Methods

### Patients

We investigated 295 transplant-eligible newly diagnosed MM (NDMM) patients **(Table 1)** and 72 cases with monoclonal gammopathy of undetermined significance (MGUS). All NDMM patients were treated with novel agents and at least one autologous stem cell transplantation. Conventional response was assessed according to the IMWG criteria 8. The study was performed under University of Arkansas for Medical Sciences IRB approval (#205415), and all patients signed written consent in accordance with the Declaration of Helsinki.

### Functional Imaging

DW-MRI was performed on a 1.5 Tesla Philips Achieva scanner as described previously 9. Briefly, the protocol included scanning from vertex to toes in 7-9 slabs depending on patient height. Each slab constituted 50 slices 5 mm thick, field of view 450 mm, matrix 112x79, repetition time 7500 ms, time to echo 69.9 ms, number of acquisitions 2, with a "Q" body coil, *b*=0 and 800 s/mm^2^. A coronal whole body *T*_1_ turbo-spin echo image was obtained as a localizer. Asplenia was excluded using coronal *T*_1_ weighted whole body survey images. Next, the spleen signal was assessed on inverted greyscale b800 DWI and exponential ADC (eADC) maps by two experienced investigators in consensus read. Absence of spleen signal was defined as (1) significant signal loss on the DWI (b800) image with isointensity to fat tissue, and (2) lack of spleen visualization on the corresponding EADC map. ADC values were not considered for this study.

For anti-CD66 antibody scintigraphy, the murine anti-CD66 monoclonal antibody BW 250/183 (anti- Granulocyte®, Scintec Diagnostics, Zug, Switzerland) was labelled with 99mTc. Planar whole body imaging using a dual-head γ-camera (Symbia Intevo, Siemens Healthineers, Erlangen, Germany) was performed 4 h and 24 h after iv injection of 650-700 MBq of the radiolabeled antibody. Low-energy, high-resolution collimators were used. A 15% energy window was centered over the 140-keV photopeak of 99mTc. After the acquisition of the 24h-planar scans, an additional SPECT/CT emission/transmission study of the abdomen was performed (Symbia Intevo, Siemens Healthineers, 180-degree acquisition per detector, 256 x 256 matrix, 3-degree steps, and 20 s/frame; CT: 110 kV, 35 mAs). Images were reconstructed using an iterative ordered subsets expectation maximization algorithm (Flash 3D: 6 subsets, 6 iterations), both with and without CT-based attenuation correction.

### Molecular analyses and risk stratification

Molecular characterization included fluorescence *in-situ* hybridization (FISH), and gene expression profiling (GEP) of CD138-enriched plasma cells (PCs) 10. Differential gene expression was performed using a threshold of 2-fold difference as described previously 11. GEP70-based risk designation and determination of the ISS were performed as described 12, 13.

### Statistics

The Kaplan-Meier method was used for survival analyses. PFS time was measured from start of treatment to relapse or death from any cause or censored at the date of last contact. Wilcoxon and Fisher's exact tests were used to compare the median of a continuous variable or the distribution of discrete variables across groups, respectively. Main analyses were undertaken using R (v3.3.1) software.

## Results

### Lack of spleen signal on DW-MRI is associated with tumor load

**Figure 1:**
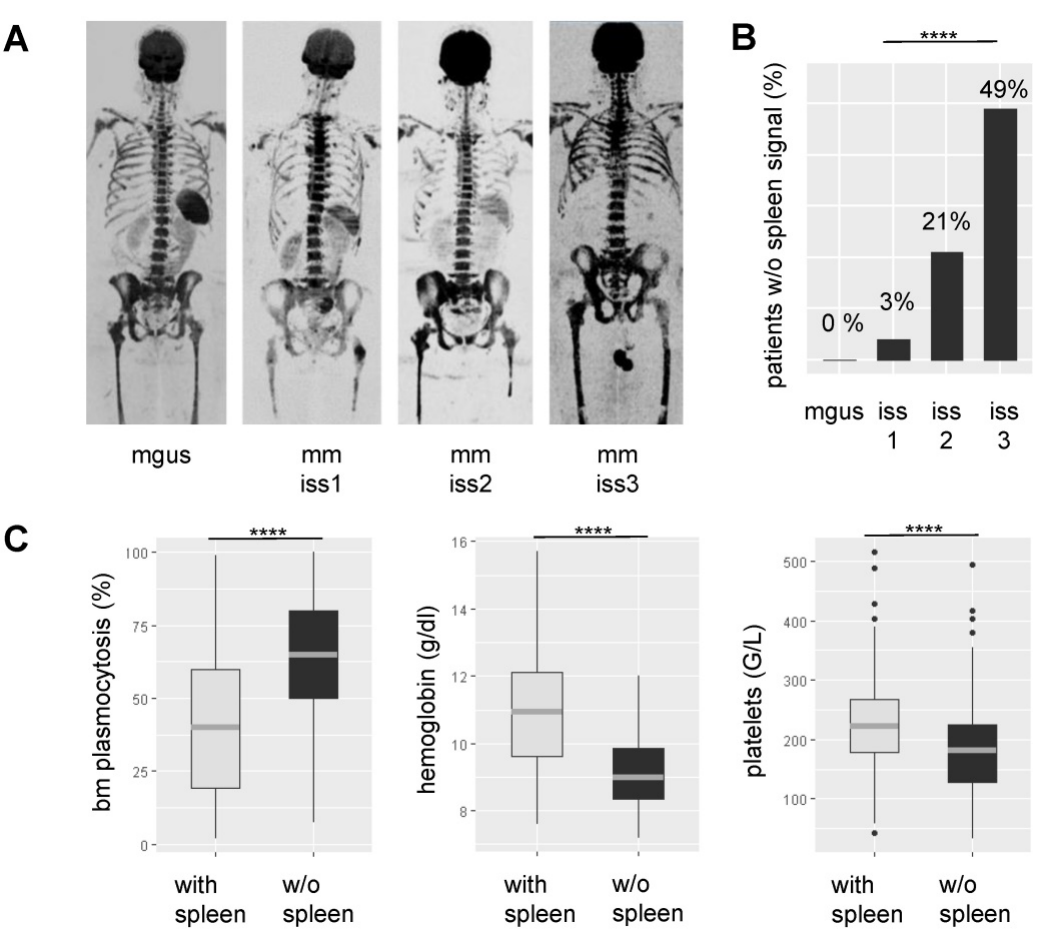
** Association between spleen signal on DW-MRI, tumor load, and hematopoietic insufficiency**. In** A** representative inverted greyscale b800 DW-MRI images for progressing stages of plasma cell disorders are shown. While the individual with monoclonal gammopathy of undetermined significance (MGUS) presents with a hyperintense spleen, the spleen signal gradually decreases and is absent in the multiple myeloma (MM) patient with International Staging System (ISS) 3. **B** shows the proportion of patients without spleen signal within progressing stages of the disease. In **C** the association between spleen signal and laboratory parameters is depicted. BM: bone marrow; **** *P*< 0.0001

Lack of spleen signal on DW-MRI was common in NDMM, seen in 71/295 (24%) patients. To elucidate the underpinnings of this observation, we correlated it with various clinical and molecular parameters. Lack of signal was highly positively associated with tumor-load parameters, such as the degree of BMPC infiltration as assessed by histology (*P*<0.0001) and International Staging System (ISS) stage 3 (*P*=0.0001) **(Figure 1)**. Apparently, it also indicated hematopoietic insufficiency, since patients without spleen signal had lower counts for hemoglobin (*P*<0.0001) and platelets (*P*<0.0001). In contrast, it was not significantly associated with age, gender, or the tumor progression markers gain(1q) and del(17p). In patients with lambda light chain MM, we observed a weak association between absence of spleen signal and increased serum free lambda light chain levels (*P*=0.03). Yet, adjusting for tumor load using the ISS stage as surrogate, there was no significant association. For kappa light chains, we did not see an association. The same holds true for the distribution of kappa and lambda light chain MM between patients with and without spleen signal. Using global gene expression data, we found no differentially expressed genes in MM cells between the two groups of patients. Together, our observations suggest that the absence of spleen signal is mainly associated with parameters reflecting tumor burden or hematopoietic insufficiency rather than with specific tumor features.

### The spleen signal is present in MGUS and reappears in MM patients during treatment

**Figure 2:**
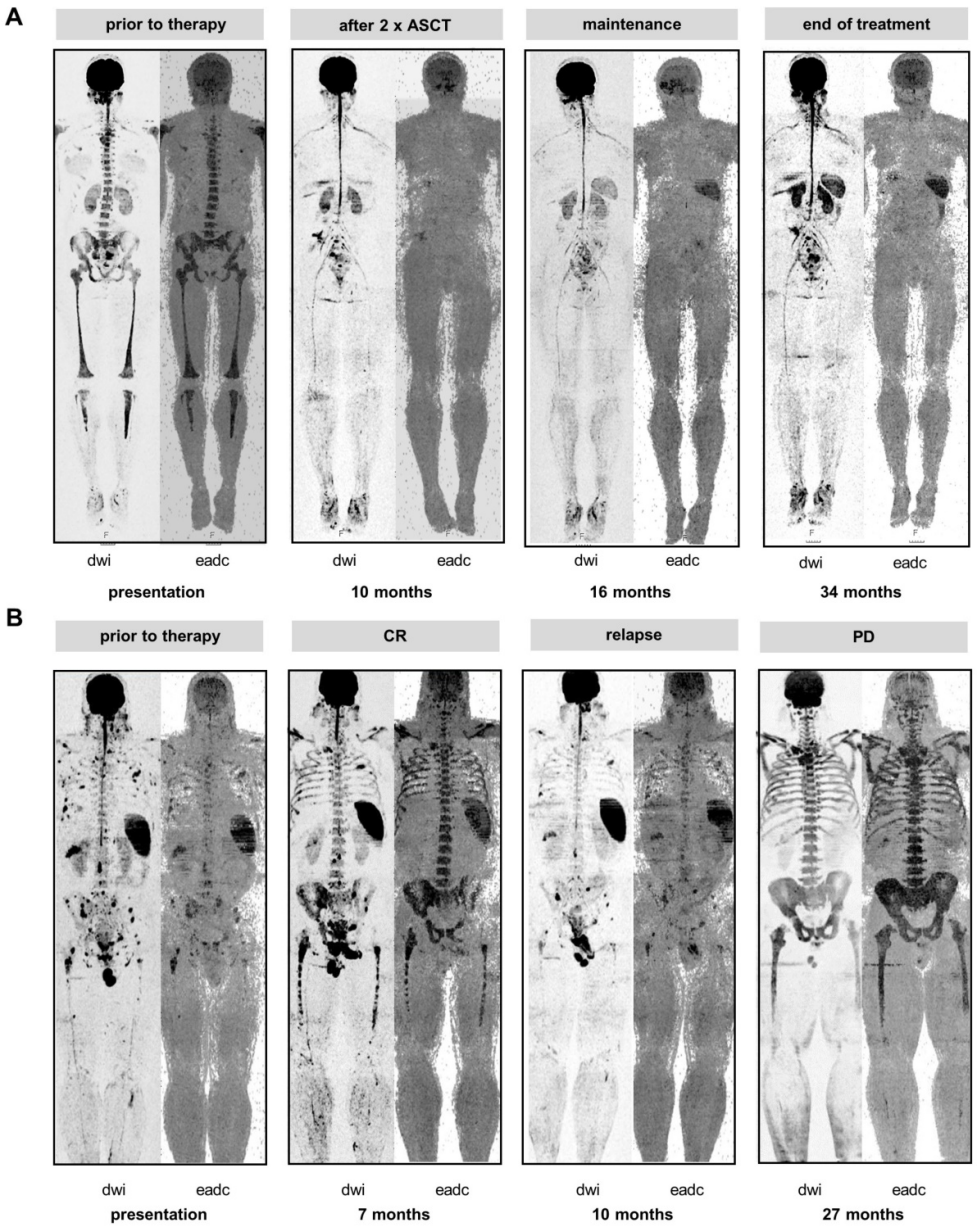
** The spleen signal during therapy. A** Baseline and follow-up inverted greyscale b800 DW-MRI images and EADC maps are shown for one patient who initially presented w/o spleen signal but experienced re-appearance of the signal during therapy. **B** Loss of spleen signal during the course of the disease. The patient relapsed with multiple small focal lesions. Subsequently, the patient experienced progressive disease (PD) and developed a severe diffuse background pattern with concomitant loss of the spleen signal.

To further address the association between the spleen signal and tumor load we hypothesized that individuals with MGUS should consistently present with preserved spleen signals due to the low level of PC infiltration in this condition. Therefore, we investigated 72 individuals with MGUS for whom DW-MRI data were available at our center. Indeed, in all MGUS cases the spleen signal was present. In addition, we monitored the spleen longitudinally in MM patients with absence of signal at diagnosis. The majority of these patients showed re-appearance of the spleen on DW-MRI as the tumor burden declined during treatment (follow-up scans available for 69 patients, of those re-appearance in 57 (83%), example shown in** Figure 2A**). In these patients the mean hemoglobin level increased from 9.2 to 11.3 g/dl (baseline vs. time point of re-appearance; *P*<0.0001). Yet, the time point of re-appearance varied between patients. For 67 patients DW-MRI data was available within 3 months after the first ASCT, and we observed re-appearance of the spleen signal in 27 (40%). These 27 patients showed deeper responses according to IMWG than patients without (≥ very good partial remission 85% vs. 45%, *P*=0.002). We also observed a trend for improved outcome in these patients (median PFS: 2.4 years vs. 5.5 years, *P*=0.07, median OS: not reached vs 5.8 years, *P*=0.07).

Vice versa, we also observed loss of spleen signal during the course of treatment. **Figure 2B** shows a series of DW-MRI images for a patient who initially presented with multiple focal lesions and a clear spleen signal. At relapse from CR, multiple small focal lesions were detectable. Subsequently, the patient developed a severe diffuse background pattern with concomitant loss of the spleen signal, which is in line with high tumor burden diminishing the spleen signal. Of note, the diffuse BM signals at progressive disease and at the prior CR were similar, illustrating that intensity of the diffuse signal was not a good proxy for tumor infiltration during treatment. In contrast, in this patient the spleen signal was useful to discriminate between malignant and non-malignant/ reactive BM hyperintensities.

### Absence of spleen signal in high risk patients heralds dismal outcome

**Figure 3:**
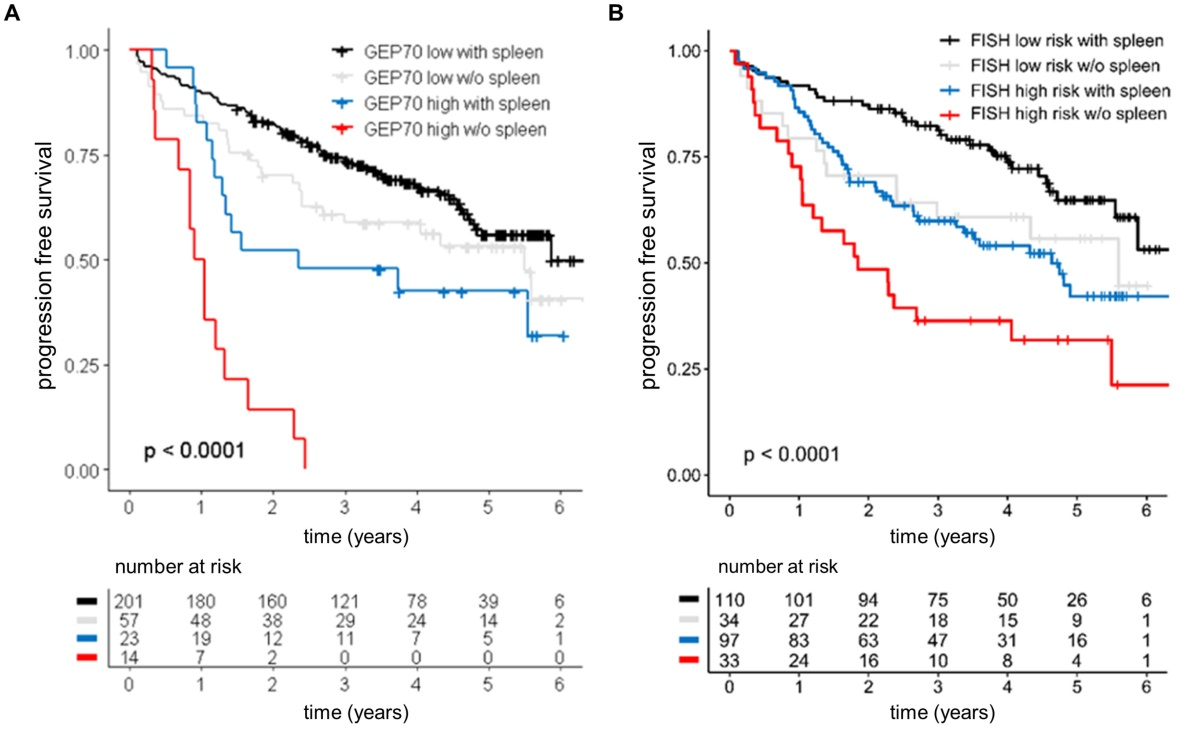
** Prognostic value of the spleen signal at baseline**. Outcome of 295 NDMM patients enrolled into Total Therapy trials stratified by the spleen signal and (**A**) the GEP70 risk score or (**B**) risk according to FISH markers. FISH high risk was defined as presence of at least one of the following parameters: t (4;14), t(14;16), del(17p), or gain(1q). The log-rank test was used to perform the group comparison.

To further investigate the prognostic value of the spleen signal we performed a survival analysis using loss of the spleen signal as categorical variable. Patients with absence of spleen signal at baseline experienced unfavorable outcome (hazard ratio of 1.8 and p<0.05 for PFS and OS) in univariate analysis. Accounting for ISS, absence of spleen signal only showed a trend towards poor PFS (p=0.06), suggesting that the negative impact in the univariate analysis was due to the strong association between the spleen signal and tumor load. Yet, we observed an interaction between the GEP70 risk status and the spleen signal. While absence of spleen had no significant impact in GEP70 low risk patients, it defined a subgroup of GEP70 high risk patients with dismal outcome (median PFS: 0.97 years, HR: 2.96, **Figure 3**). The interaction term remained significant in a multivariate analysis including ISS, showing that the spleen signal can provide prognostic information independent from ISS in GEP high risk patients. Furthermore, dismal outcome could also be seen in patients without a spleen signal and presence of at least one of the high risk chromosomal aberrations t(4;14), t(14;16), gain(1q) or del(17p) (**Figure 3**).

### The asplenia phenomenon and extramedullary hematopoiesis

**Figure 4:**
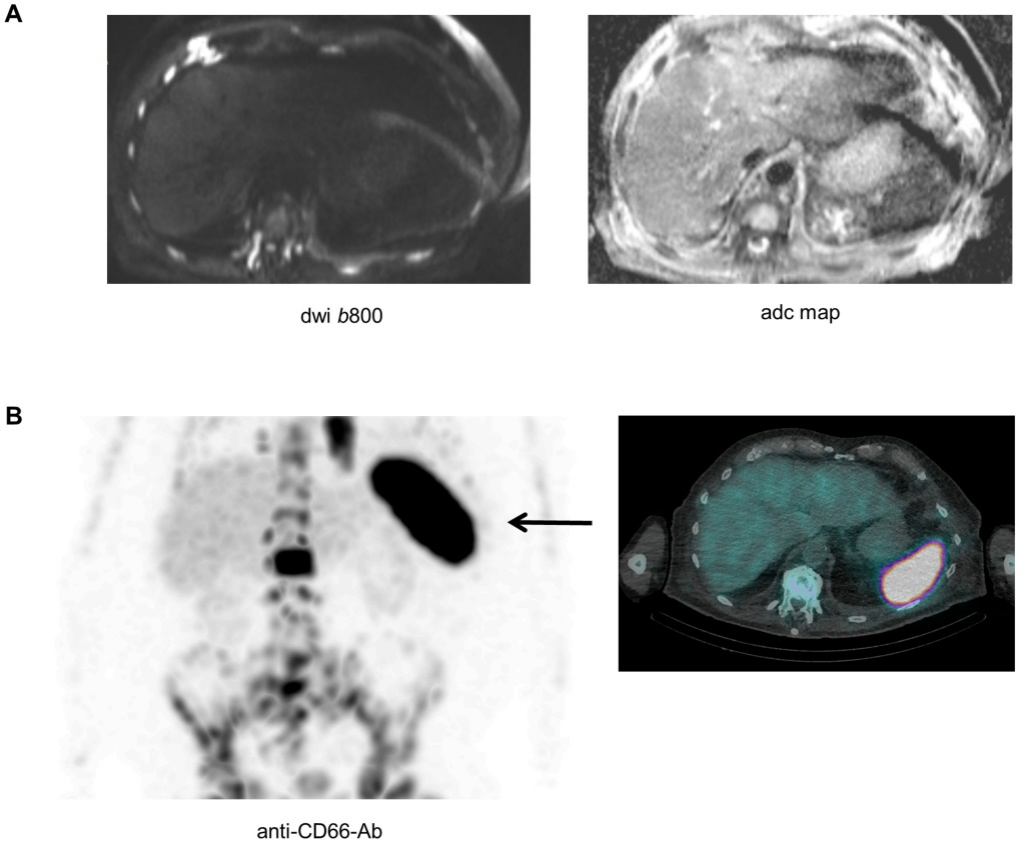
** The asplenia phenomenon and extramedullary hematopoiesis.** A patient with advanced MM and absence of spleen signal on DW-MRI also underwent Tc-99m labelled anti-CD66 antibody scintigraphy. In (**A**) the b800 DWI and ADC maps are shown. In (**B**) results of the Tc-99m labelled anti-CD66 antibody scintigraphy are depicted.

Biopsying the spleen is risky and barely possible, and as a result it is difficult to directly investigate the pathological features of the spleen in patients with absence of the signal. Yet, as recently shown, a combination of modalities could improve our understanding of the biology underlying imaging phenomena 11. A late stage relapsed patient with high tumor burden (>80%BMPC), a ferritin value of 292 µg/l (reference 30-400 µg/l), and absence of spleen signal on DW-MRI also underwent Tc-99m labelled anti-CD66 antibody scintigraphy for evaluation of a targeted radiotherapy approach. The scintigraphy revealed extensive CD66 expression in the spleen (**Figure 4**). CD66 is expressed on mature myeloid cells including promyelocytes and granulocytes 14, and was recently used as a marker for extramedullary hematopoiesis (EMH) in a patient with myelofibrosis 15. Thus, our preliminary observation suggests that EMH in the spleen is a factor that modifies the DW-MRI signal of this organ.

## Discussion

In this study, we show for the first time that the restriction level of the spleen, an organ which is frequently overlooked during myeloma examinations, is a promising proxy for tumor load in MM and also associates with response and prognosis in newly diagnosed patients. While this is an unexpected finding, we appreciate that there are better methods and parameters for assessment of tumor load, risk prediction and therapy response. Yet, we see some clinical use for the spleen signal, further increasing the value of DW-MRI as a diagnostic tool in MM 9, 11, 16-18. First, despite its strong association with the ISS, absence of spleen signal has independent prognostic value: patients with high risk disease according to a GEP signature or FISH markers suffered from dismal outcome when the spleen was not detectable. Thus, as we move forward in integrating molecular data and functional imaging in the routine work up of patients with MM, we report on a clinically relevant finding which allows for identification of a subgroup with a particularly poor outcome. Secondly, our longitudinal data illustrates that the spleen signal could be used to improve discrimination between malignant and reactive DW-MRI hyperintensities. We performed a qualitative analysis (spleen signal present: yes/no) since our scan protocol had been optimized for reproduction of FLs. Yet, as shown in Figure 1, the intensity of the spleen gradually decreased from MGUS through the ISS stages. Therefore, we postulate that the relationship between spleen signal and tumor burden could even be better described with ADC values determined using more sophisticated scan protocols.

While our observations strongly support the hypothesis, that the spleen signal is associated with a high BMPC involvement, the biological mechanism for this phenomenon remains largely elusive as it would require spleen biopsies. Yet, using anti-CD66 antibody scintigraphy in one patient with absence of spleen signal on DW-MRI, we observed a strong CD66 signal in the spleen, suggesting EMH to underlie the asplenia phenomenon in this patient. In principle, presence of EMH in patients with an obliterative BMPC involvement is not an unexpected finding, as MM cells crowd out other hematopoietic cells in the BM. In line, EMH in NDMM has previously been described by Seo *et al*., who performed a biopsy of a paravertebral mass with mild FDG uptake and detected EMH in this mass 19. EMH in MM patients was also reported by Baldane *et al.* 20 and Palma JA *et al.* 21.

Interestingly, Sato and colleagues observed hematopoietic cells solely in a tiny gap between the capsule and the amyloid protein-deposited spleen in a case of MM complicated by amyloid light chain (AL) amyloidosis with a traumatic splenic rupture after autologous stem cell transplantation 22. In this context, infiltration of the spleen with amyloid proteins, primarily throughout the red pulp, has been identified in up to 41% of cases of systemic amyloidosis 22. Since AL amyloidosis can present with diffuse low *T_2_* MRI signal of the spleen 23, the DW-MRI signal of the spleen could also be altered in these patients. Although none of our NDMM patients was diagnosed with concomitant AL amyloidosis, we cannot rule out that light chain deposition impacted the spleen signal in our study. It is also conceivable that MM PCs directly modified the spleen signal by infiltrating this organ: a study by Oshima *et al.* showed that up to 30% of autopsied MM patients presented with splenic involvement 24. Finally yet importantly, the magnetic susceptibility effect resulting from iron overload in the spleen due to red blood cell (RBC) transfusions is another factor that potentially modified DW-MRI findings in our study. However, from our clinical experience, extensive RBC transfusions in NDMM patients are uncommon.

## Conclusions

We have observed an intriguing imaging phenomenon which is strongly associated with high tumor burden and hematopoietic insufficiency, and provides prognostic information in MM. Our preliminary investigations suggest EMH in the spleen to impact the DW-MRI signal but this needs to be confirmed in future studies.

## Figures and Tables

**Table 1:** Patients' characteristics

Characteristics	No. of Patients (%)
	Total (N=295)
**Age**	
> 65 years	107 (36)
**Sex**	
Male	182 (62)
**Myeloma type**	
IgG	179 (61)
**ISS**	
I	77 (26)
II	140 (47)
III	78 (26)
**GEP70**	
low	258 (87)
high	37 (13)

## Abbreviations

ADC: apparent diffusion coefficient
BM: bone marrow
CR: complete remission
DW-MRI: diffusion weighted magnetic resonance imaging
FDG: ^18^F-Fluorodeoxyglucose
FISH: fluorescent in situ hybridization
GEP: gene expression profiling
IMWG: international myeloma working group
ISS: international staging system
MGUS: monoclonal gammopathy of undetermined significance
MM: Multiple Myeloma
NDMM: newly diagnosed MM
PET-CT: positron emission tomography computer tomography
PC: plasma cells
PD: progressive disease
